# P-1152. Staphylococcus aureus colonization at four body sites in 844 adult inpatients shows variation by demographic characteristics

**DOI:** 10.1093/ofid/ofaf695.1346

**Published:** 2026-01-11

**Authors:** Carly Siciliano, Maeve Hiehle, Brooke M Talbot, Katrina Hofstetter, Leigh Cressman, Timothy D Read, Michael Z David

**Affiliations:** University of Pennsylvania, Ardmore, Pennsylvania; University of Pennsylvania, Ardmore, Pennsylvania; Emory University, Atlanta, Georgia; Emory University, Atlanta, Georgia; University of Pennsylvania Perelman School of Medicine, Philadelphia, Pennsylvania; Emory University School of Medicine, Atlanta, Georgia; University of Pennsylvania Perelman School of Medicine, Philadelphia, Pennsylvania

## Abstract

**Background:**

*S. aureus* (SA) colonization in nares is common, but few studies have examined prevalence of and risk factors for extra-nasal colonization in hospital inpatients. Little is known about hand colonization in this population.

**Methods:**

844 inpatient adults on two general medicine hospital units were tested for SA colonization of nose (N), throat (T), groin (G), and hands (H) in 12/2023-3/2025. Samples were obtained within 3 days of unit admission and processed by direct plating and broth enrichment if direct plating was negative. N, T, and G cultures were obtained by dry rayon swabs. H was tested by phosphate-buffered saline wash. Demographic and clinical data were abstracted from the electronic medical record.

**Results:**

44.1% (372/844) had colonization at one or more body sites (Table 1); 32.9% had N, 25.0% T, 18.3% G, and 29.6% H colonization. Multi-body-site colonization was common; of nose-colonized subjects, 55.8% had T, 46.0% had G, and 72.3% had H carriage. Subjects had sole colonization in N (n=49, 5.81%), T (n=33, 3.9%), G (n=8 1.0%), and H (n=26, 3.1%); if these sites were not assessed colonization would have been undetected.

Of subjects with SA, 25.3% (or 11.1% of the entire cohort) had exclusive extra-nasal colonization. If H colonization is disregarded, 18.3% of SA carriers (or 8.1% of the entire cohort) had exclusive extra-nasal colonization.

Demographics and a comparison of subjects with and without colonization at any one or more sites are shown (Table 1). Colonization was more common in each N, G, and H (but not T) in men than women. Those colonized at each individual site were younger. Medicaid subjects had higher SA prevalence at each individual site than with other insurance. White subjects were more likely carriers at T (but not other sites) than others. Subjects with a clinical SA isolate cultured in hospital were more likely to have SA at N (p=0.001), G (p=0.015), and H (p=0.05).

**Conclusion:**

SA colonization was common in adult inpatients at four body sites, particularly in younger subjects, those on Medicaid, and with a clinical SA isolate. Males and white subjects were more likely to have T colonization. Nose carriers were frequently colonized at other body sites, particularly the hand. Testing in the nares only would far underestimate true SA carriage.

**Disclosures:**

All Authors: No reported disclosures

